# The Study of Airborne Particulate Matter in Dalnegorsk Town

**DOI:** 10.3390/ijerph18179234

**Published:** 2021-09-01

**Authors:** Aleksei S. Kholodov, Irina A. Tarasenko, Ekaterina A. Zinkova, Michele Teodoro, Anca Oana Docea, Daniela Calina, Aristidis Tsatsakis, Kirill S. Golokhvast

**Affiliations:** 1 Far East Geological Institute, Far East Branch, Russian Academy of Sciences, 690022 Vladivostok, Russia; alex.holodov@gmail.com (A.S.K.); tarasenko_irina@mail.ru (I.A.T.); 2 Far Eastern Scientific Center of Russian Academy of Education, Far Eastern Federal University, 690922 Vladivostok, Russia; k762000@mail.ru (E.A.Z.); golokhvast.ks@mail.ru (K.S.G.); 3 Department of Biomedical and Dental Sciences and Morphofunctional Imaging, Occupational Medicine Section, University of Messina, 98125 Messina, Italy; michele.teodoro@unime.it; 4 Department of Toxicology, University of Medicine and Pharmacy of Craiova, 200349 Craiova, Romania; 5 Department of Clinical Pharmacy, University of Medicine and Pharmacy of Craiova, 200349 Craiova, Romania; 6 Department of Forensic Sciences and Toxicology, Faculty of Medicine, University of Crete, 71003 Heraklion, Greece; tsatsaka@uoc.gr; 7 Department of Analytical and Forensic Medical Toxicology, Sechenov University, 119991 Moscow, Russia; 8 Pacific Geographical Institute, Far East Branch of the Russian Academy of Sciences, 690041 Vladivostok, Russia; 9 Siberian Federal Scientific Center of Agrobiotechnology RAS, 630501 Krasnoobsk, Russia

**Keywords:** atmospheric pollution, environmental pollution, public health, laser diffraction analysis, atmospheric particulate matter

## Abstract

Mines, quarries, dumps, and tailings are the sources of air pollution. In the Dalnegorsk District (Primorsky Krai, Russia), there are 20 polymetallic deposits. This study aimed to evaluate the particle size and material composition of ambient particulate matter (PM) in Dalnegorsk town and verify the influence of mining and chemical industry facilities on the composition of PM. Ambient particulates were analyzed in samples of snow cover and washout from vegetation (conifer tree needles). According to particle size distribution data, the relative content of particles with a diameter up to 10 microns (PM_10_) reaches 40% in three snow samples taken in the central part of the town. Among ore minerals, pyrite and arsenopyrite predominated in the samples. In addition, sphalerite, galena, cassiterite, and iron–chromium–nickel formations of various shapes were found in the studied particles. The presence of these metals in airborne PM can negatively affect the incidence rate of PM-associated diseases and the determination of their levels are very useful for air pollution prevention strategies.

## 1. Introduction

Air pollution effects on living organisms represent not only a risk factor for human and animal health but also to the environment. One of the main factors in air pollution is ambient particulate matter (PM), especially fine particles which can penetrate the human respiratory tract. Exposure to PM was linked with increased risk for pulmonary disease-associated morbidity and mortality as bronchitis, pneumonia, asthma, and other respiratory diseases [1,2,3,4], cardiac-associated morbidity and mortality [5,6], and increased risk for viral respiratory infections as COVID-19 [7,8,9,10].

Particles with an aerodynamic diameter less than 10 μm (PM_10_) are transported by air currents over long distances [11]. Fine particles (PM_2.5_) can travel larger distances and stay in the air longer [12]. Particle size, chemical composition, and the total concentration of PM are the most important parameters in assessing the impact of dust on ecosystems and humans [13,14].

Mines, quarries, dumps, and tailings are the sources of air pollution. According to I.A. Turchaninov, a gust of wind can blow about 60,000 m^3^ of sand from the surface of a 1000 hectares tailing dump [15]. Dust on the surface of dry tailing dumps contributes to the emission of fine particles containing metals into the atmosphere.

One of the oldest enterprises of Primorsky Krai—JSC MMC Dalpolymetal, which today exploits six polymetallic deposits (Nikolayevskoye, Partizanskoye, Yuzhnoye, Maiminovskoye, Silinskoye, Verkhneye) is located in the Dalnegorsk urban district, surrounded by Sikhote-Alin ridges. All of the above deposits are either located near the processing facilities or within 45 km of the processing plant, which is located in the center of Dalnegorsk. The exception is the Silinskoye deposit located 70 km from Dalnegorsk. The ores in these deposits include three general groups of minerals. The main group contains pyrrhotite, sphalerite, galena, jamsonite, siderite, quartz, calcite, and rhodochrosite; the minor group: tennantite, arsenopyrite, axinite, manganocalcite, and actinolite; the accessory group: native silver, discrasite, stannite, cassiterite, tetrahedrite, garnet, tourmaline, albite, diopside, wollastonite, and datolite [16].

There are several tailing dumps in the Dalnegorsk District which were decommissioned but not covered with protective layers.

Mature fine tailings are represented by a mixture of mineral particles ranging in size from fractions of a micron to 3 mm. The particle size distribution of soils is characterized by the predominance of pelitic (−0.002 mm, 52%), silt (−0.05...+0.002 mm, 36%), and psammitic (−2...+0.05 mm, 10%) fractions. The sum of sulfides in technogenic deposits varies from 5 to 35%. Of these, pyrite and marcasite amount to 80%, sphalerite and galena to 15%, pyrrhotite, stannite, and arsenopyrite to 4–5% [16].

The settlements closest to the tailing dumps (Dalnegorsk town and Krasnorechensky village) are located in the valley of the Rudnaya River, running between hills. The mass of dust containing metals and harmful elements influences the human environment and natural ecosystems in the area.

Directly within Dalnegorsk town, there is a borosilicate deposit developed by JSC Mining and Chemical Complex “Bor” by open pit method using drilling and blasting operations. The processing of the borosilicate mineral (datolite) results in the formation of significant quantities of unclaimed borogypsum as a waste product. The total mass of waste at “Bor” Complex is about 50 million tons, and the main components are calcium sulfate (gypsum) and finely dispersed silicon dioxide. These components make up more than 90% of the total weight [17].

This work aimed to study the particle size and material composition of ambient particulate matter in Dalnegorsk town and verify the influence of mining and chemical industry facilities on the composition of PM in order to identify the risk for air pollution in the region.

Ambient particulates were analyzed in samples of snow cover and washout from vegetation (conifer tree needles). Snow precipitation is commonly used to assess the condition of the aerial environment in urban areas [18]. Conifers, due to the structure of their needles, are effective in accumulating fine and coarse particles and can be used as bio accumulators of air pollution [19].

## 2. Materials and Methods

### 2.1. Snow Cover Samples

Snow cover samples were collected in Dalnegorsk town to analyze the particle size distribution of PM. Only the top layer of fresh snow (5–10 cm) was collected immediately after a snowfall to exclude the secondary pollution of settled snow. Snow samples were collected from a 1 m^2^ area into 2.5-L plastic containers pre-washed with distilled water. The samples were immediately transported to the laboratory.

### 2.2. Determination of Particle Size Distribution in Snow Samples

In melted snow samples, the particle size distribution was measured by the laser diffraction method. Measurement results calculated using the Mie-Gruneisen equation of state indicate the average particle size and the percentage of particles of different fractions. Particle size is expressed as the diameter of a sphere of equivalent volume. In the study, we used the Analysette 22 NanoTech plus laser particle analyzer (Fritsch GmbH, Idar-Oberstein, Germany) equipped with a measuring unit, sample dispersion units, and an AutoSampler unit. Before analysis, liquid samples were shaken, and a 40 mL aliquot was taken from each sample for analysis. The measurements were run at the settings of quartz/water at 20 °C in three repeats. The detection range was 0.008–2000 μm. The measurement results were processed automatically using the software supplied with the laser particle analyzer. Statistical analyses were performed in the software package STATISTICA 10 (StatSoft, Inc., Tulsa, OK, USA).

### 2.3. Conifer Needle Samples

Conifer needles of Yezo spruce (*Picea jezoensis*) were collected according to the previously described method [20] from trees at the height of 1–1.5 m and carefully transported to the laboratory. In the laboratory, the containers with sample needles were filled with double-distilled water and cleaned with ultrasound using a Sonopulse 3100 HD ultrasonic homogenizer (Bandelin electronic GmbH & Co. KG, Berlin, Germany) at 22 kHz, 100 watts, and a 5-min exposure to remove the dust particles from the needles. Ultrasound treatment of conifer needles was an effective method to obtain samples with increased content of particulate matter for analysis. The resulting liquid was filtered through a 0.22 μm Millipore filter and then the filter was dried in an oven.

### 2.4. Determination of PM from Dried Residue and Reference Material

Dried residue and reference material (samples of minerals and coal) were analyzed by Raman spectroscopy using the Morphologi G3-ID equipment (Malvern Instruments Ltd., Malvern, UK). Morphologi G3-ID combines the dispergation system and automated static imaging features with the chemical identification of individual particles using Morphologically-Directed Raman spectroscopy. Using this method, the size and basic chemical composition of each particle in a sample can be determined by comparing it with the spectra of reference material [21]. In each sample, 200 particles with a diameter of 5–20 µm were analyzed in manual mode and 400 particles with a diameter of 20–25 µm—in automatic mode.

### 2.5. Ore Mineral Samples

To assess the effect of fine particles of ore minerals on the overall air pollution in the town, we collected samples of ore minerals widespread in this area: datolite CaBSiO_4_(OH), danburite Ca[B_2_Si_2_O_8_], calcium-iron garnet Ca_3_FeSi_3_O_12_, galena PbS, calcite CaCO_3_, hedenbergite CaFeSi_2_O_6_, wollastonite CaSiO_3_, and sphalerite ZnS. Raman spectra of these minerals and carbon were compared with the spectra of particles in the filtered and dried washout from conifer needles by correlation analysis using the software supplied with the analyzer.

### 2.6. Imaging, Morphometry, and Microanalysis of Metal Particles

For imaging, morphometry, and microanalysis of metal particles, we used the new generation TESCAN LYRA 3 XMH microscopes (with an additional EDS AZtec X-Max 80 Standart ion column) equipped with the Oxford AZtec Energy microanalysis system. When scanning samples, we used: low and medium electron beam accelerating voltages (from 5 to 15 kV); short working distances to reduce spherical aberrations with the corresponding improvement in spatial resolution; In-Beam SE secondary electron detector designed to efficiently accumulate a useful signal even at short working distances.

For qualitative and quantitative elemental analysis, characteristic X-ray radiation (X-ray) was used due to the application of energy and wave dispersion detectors with a detection limit for most elements of 0.05–0.1% mass and accuracy of composition determination of ±1–2% (for concentrations > 10% mass).

The INCAFeature Oxford Instruments software application was used to automate the search process. DEPTH mode with extended focal depth was used for the SEM column, which is important in this case for the analysis of relief objects. The unit step size of the electron probe in automatic scanning was 200 nm. The search covered the entire surface of the sample available for analysis. All particles in which the phase concentration was above the EDS detection limit were recorded.

In addition, the elemental composition of minerals was studied using a four-channel JEOL JXA 8100 microanalyzer equipped with three wave spectrometers and an energy dispersive spectrometer INCAx-sight (Oxford Instruments, Abingdon, UK). Imaging conditions were: accelerating voltage, 20 kV; probe current, 10 nA; probe diameter, 1–2 μm. Standards used were pure elements, compounds, or minerals supplied by Micro-Analysis Consultants Ltd., Oxford, UK.

The analytical studies were carried out in the Education and Scientific Center of Nanotechnology, Far Eastern Federal University, and in the Laboratory of Micro- and Nanoresearch of Far East Geological Institute, FEB RAS.

### 2.7. Study Area

**Figure 1:**
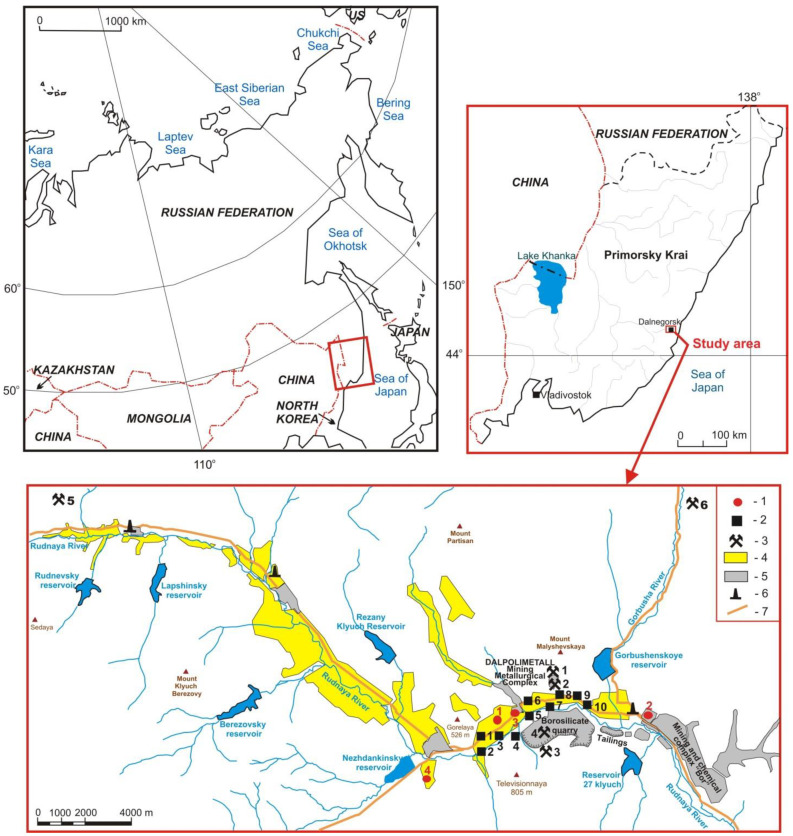
The study area and locations of vegetation (1) and snow sampling points (2); deposits under exploitation (3): 1, Nikolayevskoye; 2, Verkhneye; 3, Partizanskoye; 4, Dalnegogorskoye borosilicate; 5, Yuzhnoye; 6, Mayminovskoye; residential area (4); industrial area (5); boiler houses (6); regional highway (7).

The samples of snow cover and vegetation were collected in Dalnegorsk town at sampling points specified in Figure 1 and Table 1.

## 3. Results

### 3.1. Snow Cover Studies

**Figure 2:**
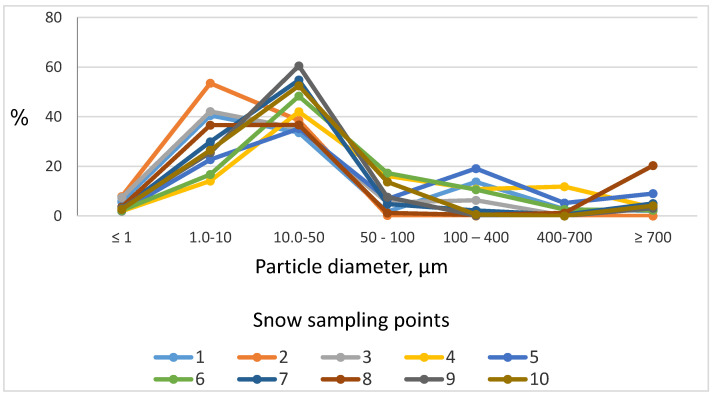
Particle size distribution of particulate matter measured in melted snow samples from Dalnegorsk town.

The size distribution of particles in melted snow samples is presented in Table 2 and Figure 2. In Dalnegorsk, high content of PM_10_ particles (more than 40% of all particles) was observed in samples from the central part of the town near the road (points No. 1–3). The background point (No. 4) located in a forest at some distance from the road, had the lowest PM_10_ content of all samples (14.1%).

### 3.2. Study of Washout from Vegetation Samples (Conifer Needles)

The results of the correlation analysis (Table 3) show the relative content of minerals widespread in the study area and coal in conifer washout samples (in %).

According to the obtained data, calcite predominated in all studied samples. Coal particles were found in all samples. In sample No. 2, the coal fraction reached 22%, which was caused by the influence of the boiler house of the “Bor” enterprise, which has coal and oil-burning boilers. In the same sample, 3.4% of galena particles were found. Furthermore, silicate particles were present in all samples. Interestingly, only in the background sampling point located in a forest on the outskirts of a private sector (No. 4), sphalerite particles were found (8.4%). It should be noted that this type of study is limited to the materials selected for correlation analysis. In reality, the composition of microparticles is much more diverse.

**Figure 3:**
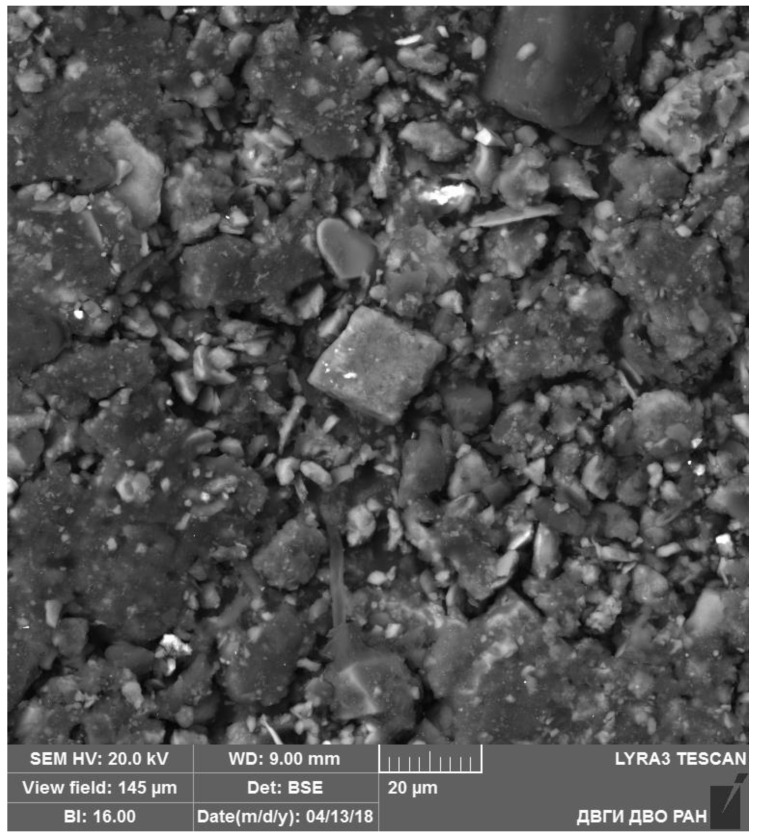
General view of a dust washout sample.

The scanning electron microscopy of finely dispersed phases of dust washout from vegetation confirmed the earlier observations. This method also showed that dust particles included minerals (silicates, phosphates, chlorides, and sulfides) (Figure 3).

**Figure 4:**
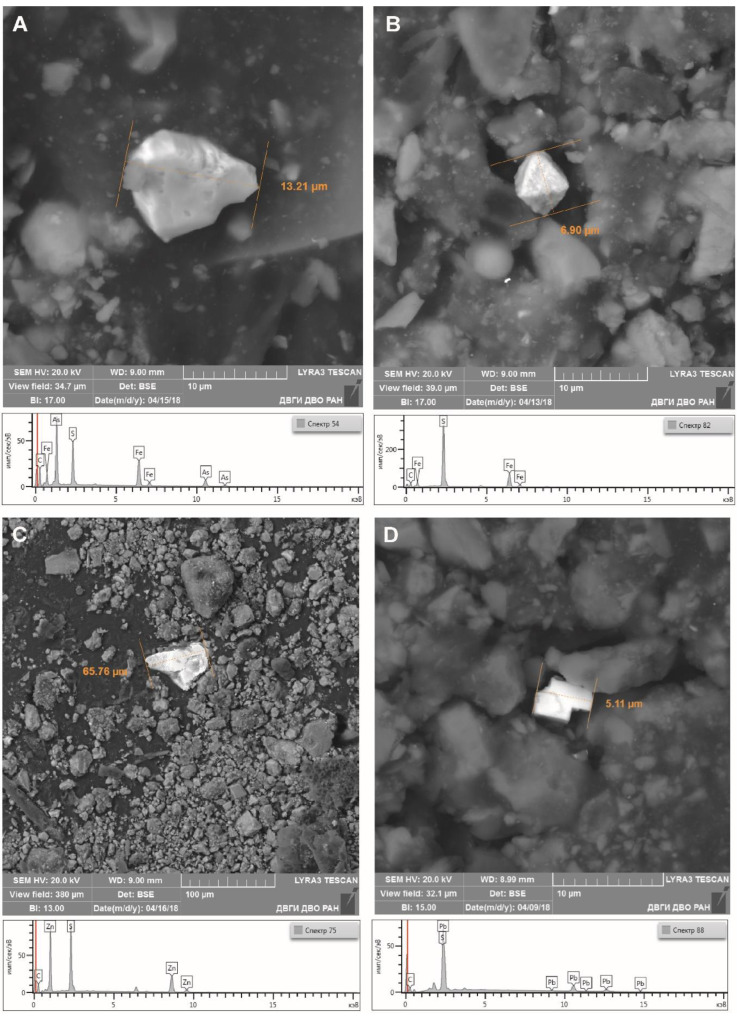
Morphology and chemical composition of Fe, As, Zn, Pb, and S dust particles represented by: (**A**) arsenopyrite (washout sample No. 3); (**B**) marcasite (sample No. 2); (**C**) sphalerite (sample No. 3); and (**D**) galena (sample No. 1).

According to the data of scanning electron microscopy, the main components in the samples were Fe, As, and S concentrated in finely dispersed phases of ore minerals, pyrite (FeS_2_) and arsenopyrite (FeAsS) (Figure 4A), and also microcrystals shaped as tetragonal dipyramid, which are, presumably, marcasite (FeS_2_) (Figure 4B). The dust washout also included particles containing Zn and Pb represented by morphotypes of such ore minerals as sphalerite (Figure 4C) and galena (Figure 4D).

**Figure 5:**
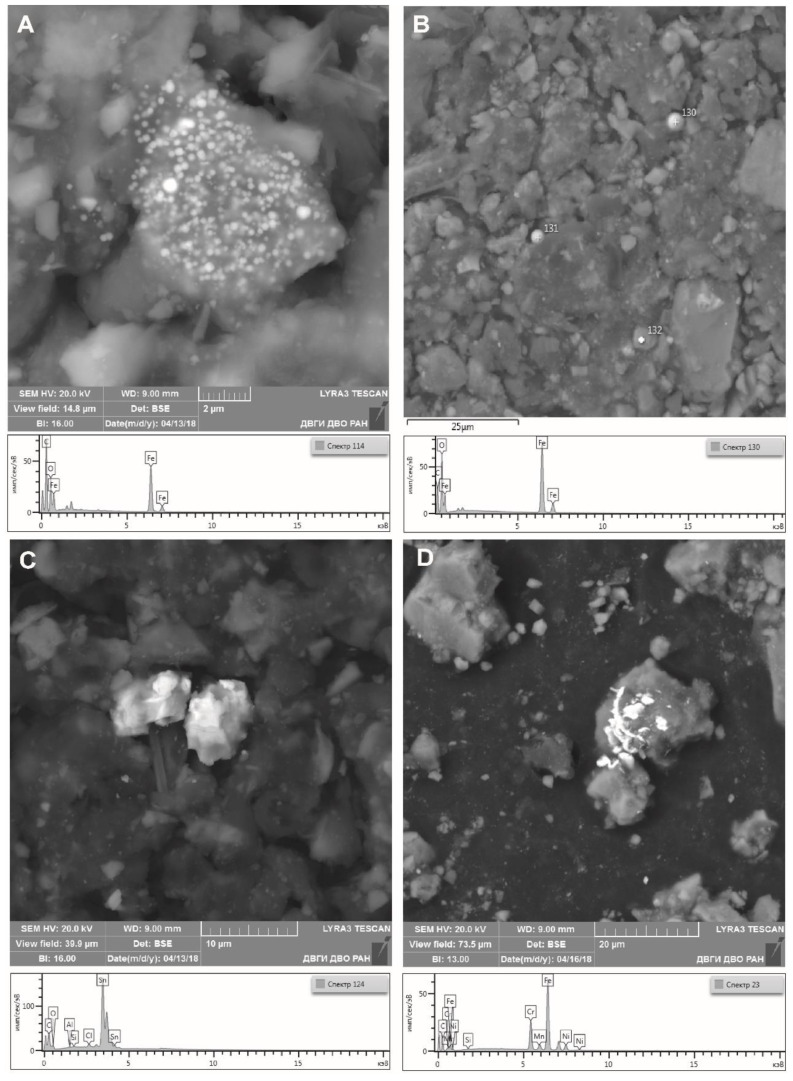
Morphology and chemical composition of Fe, Sn, Cr, and Ni dust particles represented by: (**A**,**B**) magnetite (sample No. 2); (**C**) cassiterite (sample No. 2), and (**D**) iron-chromium-nickel minerals (sample No. 3).

In addition, the studied samples contained clusters of spherical particles represented by iron oxide, probably magnetite (Figure 5A,B), and also cassiterite (SnO_2_) containing up to 86% tin (Figure 5C) and iron-chromium-nickel formations of various shapes (Figure 5D).

**Figure 6:**
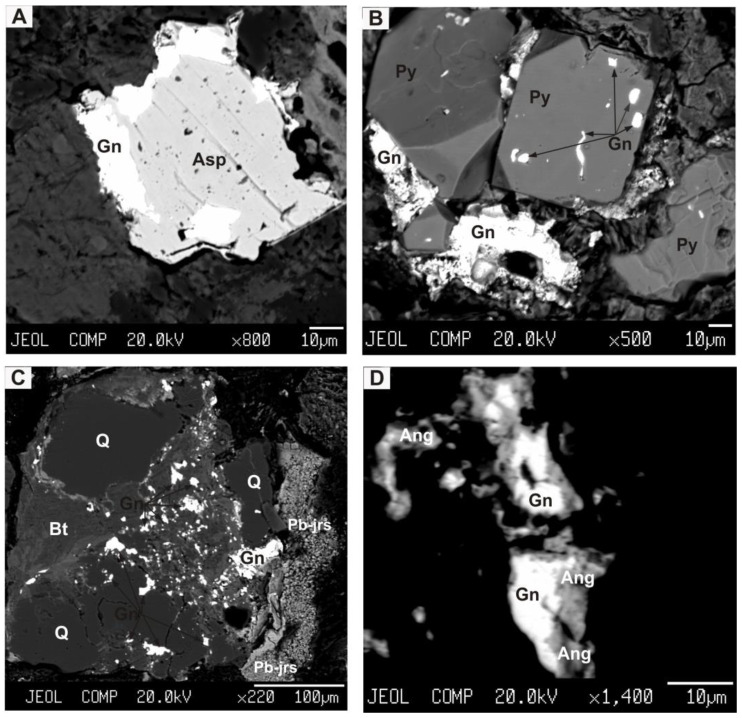
Relationship of minerals in dust particles: (**A**) galena (Gn) and arsenopyrite (Asp); (**B**) galena (Gn) and pyrite (Py); (**C**) galena (Gn), plumbojarosite (Pb-jrs), quartz (Q) and biotite (Bt); (**D**) galena (Gn) and anglesite (Ang). Photographs in backscattered electrons.

Lead is a component of galena (51.63% of the total volume of lead) and cerrusite (35.3%). It was found in small amounts in plumbojarosite (11.11%) and anglesite (1.96%) (Figure 6). Tin is a part of cassiterite (93.6% of the total volume of Sn) and stannine (4.3%). Zinc is found mainly in marmatite (49.54%). Marmatite also contains 0.12% cadmium, 0.06% indium and 0.08% copper.

## 4. Discussion

Typically, the increased content of PM in urban areas is associated with traffic [22]. However, in the case of Dalnegorsk town, the results needed verification due to the town’s specific geochemical background. According to the data in this study, fine particles of dust containing the studied elements are carried over the entire territory of Dalnegorsk town with the airflow. In Dalnegorsk, eastern and western winds prevail throughout the year, carrying airborne PM along the central street stretching from east to west.

Thus, using a complex research technique, it was possible to determine that the ambient air of Dalnegorsk town contains microparticles (PM1, PM2.5, PM10) with metals that are classified as hazardous (Pb, Zn). These metals are present mainly in the composition of ore minerals, which is typical for the atmospheric background of the Dalnegorsk urban district. In addition, the samples contained coal, the sources of which are boiler houses.

The climate (long cold winter), low standards of living, and environmental aspects are cited as the main factors affecting the incidence of diseases of the respiratory system, skin, and circulatory system of the Dalnegorsk population [23]. According to the medical statistics, in the Dalnegorsk district, there is a high incidence of respiratory diseases in all age groups of the population and a very high incidence of skin diseases [23]. The number of cancer pathologies is high as well. In Dalnegorsk, as in many other settlements in the Primorsky Krai, a direct correlation was found between the so-called “environmental stress zones” and the incidence of lung cancer, stomach cancer, skin cancer, intestinal cancer, and bladder and kidney cancer [24].

In a study of hygienic aspects of respiratory diseases in the population of Primorsky Krai’s industrial centers, the composite index of man-induced impact on the population in Dalnegorsk was 0.609 points, exceeding the same index calculated for other settlements of Primorsky Krai [25]. The integral exposure index is an indicator that allows estimating the impact on the human body of every single environmental factor that contributes to the development of eco-dependent pathologies, as well as reliable assess the body’s response to environmental influences. The lower the index, the worse the environmental situation in the area [26]. This value represents particularly unfavorable environmental conditions in Dalnegorsk, contributing to an increase in the incidence of respiratory diseases.

According to the official statistics, the average long-term incidence of respiratory diseases in children and adolescents in Dalnegorsk town exceeds the regional level by more than 1.5 times. In 2019, the primary disease incidence of the adult population with chronic unspecified bronchitis and emphysema in Dalnegorsk exceeded the average regional level by 2.1 times [27].

In addition, the long-term incidence of endocrine system diseases, nutritional disorders, and metabolic disorders in children, adolescents, and adults in Dalnegorsk exceeds the average regional level by 1.5 and more times [27].

According to the Regional Program of the Primorsky Krai “Cardiovascular diseases control” [27], the primary incidence of circulatory system diseases in the population of the Dalnegorsk urban district increased by 260.7 people per 100,000 of the population from 2014 to 2018, reaching 2654.61 cases per 100,000 population.

The overall mortality rate in Dalnegorsk in 2020 statistically (*p* ≥ 0.95) exceeded the average regional level by 1.32 times, reaching 20 people per 1000 population. Today Dalnegorsk ranks fourth in terms of mortality among all territories of the Primorsky Krai.

The State report on healthcare in the region points to malignant neoplasms as the main factor in the mortality of the population [27]. According to Kiku et al., [24], the average cancer incidence in Dalnegorsk in 2000–2014 was 290.2 cases per 100,000 population, placing Dalnegorsk seventh among all settlements in the Primorsky Krai. In 2019, the cancer incidence index in Dalnegorsk was the most unfavorable and amounted to 534.2 people per 100,000 population. The research states that the total influence of environmental factors on the cancer incidence in Dalnegorsk reaches 27.5% [24].

There is also evidence of high content of the studied elements in anthropogenic waters of the tailing dumps that enter the Rudnaya River passing through Dalnegorsk town. According to previously obtained data, the concentrations of most of these elements are diluted by the watercourse to safe values about 500 below the site of seepage from the tailings dump [28].

**Figure 7:**
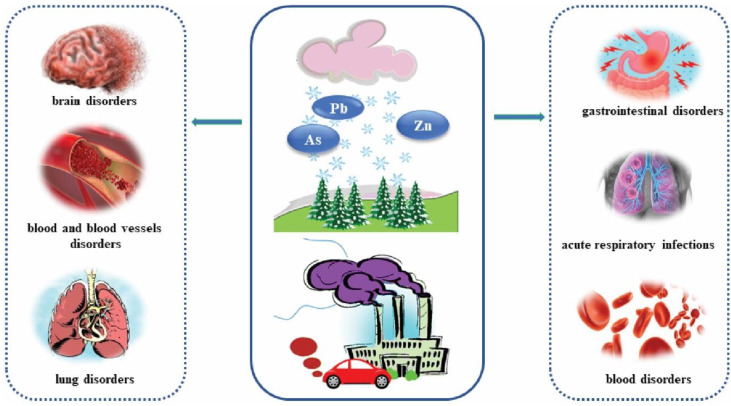
Diagram with the most important harmful effects of pollution particles on human health.

The impact of the majority of these elements in high concentrations found in highly polluted regions on human organisms leads to harmful consequences (Figure 7).

Zinc is a mutagenic, gonadotoxic and embryotoxic element. Long-term exposure to its compounds leads to gastrointestinal disorders, an increase in the number of acute respiratory infections, dental caries, changes in the morphological composition of the blood, and an increase in the overall incidence of children [29]. In addition, an excess of zinc causes dead spots to appear on plant leaves [30].

Lead is a polytropic poison that causes pathological changes in the nervous system, blood and blood vessels. It causes lung diseases in children. Inorganic lead compounds (Pb^2+^) disrupt the body’s metabolism and inhibit blood and tissue enzymes. Lead can enhance the development of anemia diseases. Diseases of the peripheral nervous system (cephalalgia, ishalgia, myalgia) are associated with an excess of lead in soils and waters. Lead accumulates in bones, hair, tissues of the aorta, liver, and kidneys, causing paralysis of the nervous system, anemia, and intestinal spasms [31]. In the vicinity of polymetallic mines, there is often a direct correlation between the distribution of lead in soils and the incidence of arteriosclerosis and tooth decay [32,33].

Arsenic and its compounds are highly toxic. They affect the nervous system, vascular walls, increase capillary permeability and paralysis and develop necrobiotic lesions in the liver, kidneys, heart, and intestines [34,35]. In addition, they cause disorders of fat and hydrocarbon metabolism, dermatitis in children, and skin cancer [34]. High concentrations of arsenic in soils lead to plant death.

For the environmental pollution and public health issues is very important to evaluate the level of PMs in highly industrialized regions and to use bio accumulators for this assessment as conifer needles. The levels of PM in the environment can provide new evidence for the regulators to impose new rules for the industry in order to prevent public health risks, especially in a vulnerable population.

## 5. Conclusions

Air pollution in Dalnegorsk town has its own specific aspects due to the geographical location of the town and the geochemical specifics of the region. In addition to standard stationery and mobile sources of pollution (boiler houses, motor vehicles, etc.), the composition of airborne PM and ultimately the pollution of the human environment is influenced by mine tailings represented by a mixture of mineral particles of different sizes (from a fraction of a micron to 3 mm) and composition.

Application of the highly localized analysis and visualization techniques made it possible to determine the presence of fine grains of varied composition in the samples. The studied particles of ore minerals are characteristic of the geochemical background of the Dalnegorsk urban district, where large polymetallic and borosilicate deposits, mining, and processing enterprises are located. This determines the composition of dust containing hazardous elements (Fe, As, Zn, Pb, S, Ni, and Cr), which have a negative impact on the environment and human health.

## Figures and Tables

**Table 1:** Description of sampling points in Dalnegorsk town.

Sampling Point No.	Description of Sampling Point
Snow cover sampling points	
1	Pionerskaya Str., snow sampling point near the stadium
2	Residential area on 50 Let Oktyabrya Ave., 20 m from the road
3	Residential area, sampling point on Polina Osipenko Blvd
4	Forested area, background point
5	Park area
6	Sampling point near a private residential building on 6 Sovetskaya Str
7	Front yard of a building on 50 Let Oktyabrya Ave. 15 m from the road
8	Private residential building on Sovetskaya Str
9	Country road
10	Private residential building on 50 Let Oktyabrya Ave. 15 m from the road
Vegetation samples (conifer needles)	
1	Town center, sampling point near the house on 64 Pionerskaya Str
2	Near “Bor” enterprise, 30 m from the road on the outskirts of the town
3	City center, 10 m from the road
4	The southern end of town, outskirts of the private sector. Background point in the forest on a hill, 550 m from the road

**Table 2:** Particle size distribution (in %) of particulate matter measured in melted snow samples from Dalnegorsk town.

Sampling Point No.	Diameter μm						
	≤1	1–10	10–50	50–100	100–400	400–700	≥700
	Percentage of Fraction (Distribution by Number)						
1	5.8	40.5	33.6	1.7	13.7	2.7	2
2	7.8	53.5	38.5	0.2	0	0	0
3	7.4	42.1	35.2	5.4	6.3	0.1	3.5
4	1.9	14.1	42	16.1	10.8	11.8	3.3
5	2	22.7	35.2	6.8	19.1	5.2	9
6	2.2	16.7	48.3	17.3	10.6	2.6	2.3
7	2.8	29.9	54.8	4.8	2.2	0.5	5
8	3.6	36.6	36.7	1.2	0.4	1.2	20.3
9	3.2	25.5	60.5	7.6	0.1	0	3.1
10	2.6	26.5	52.5	13.7	0.6	0	4.1

**Table 3:** Software chemical correlation based on Raman spectra of particles (5–25 µm; n = 600 particles in each sample) in the samples of washout from conifer needles with material standards.

Material	The Content of the Material in Samples (Distribution by Number) %			
	Sample 1	Sample 2	Sample 3	Sample 4
Datolite	0.3	-	-	-
Galena	0.1	3.4	-	-
Calcite	86	56.3	92.1	80.3
Hedenbergite	0.5	-	0.1	0.6
Wollastonite	0.1	-	-	0.1
Sphalerite	-	-	-	8.4
Coal	4.3	22	3.6	3.1
Silicate particles	8.2	0.9	3.6	7
Calcium–iron garnet	0.1	-		0.1
Not identified	-	17.1	0.3	-

## Data Availability

The dataset presented in this study is available from the corresponding author upon reasonable request.
